# A Benign Medullary Thyroid Cancer

**DOI:** 10.7759/cureus.21038

**Published:** 2022-01-08

**Authors:** Reza Pishdad, Mona Vahidi Rad, Lissette Cespedes

**Affiliations:** 1 Division of Endocrinology, Diabetes and Metabolism, Johns Hopkins University School of Medicine, Baltimore, USA; 2 Department of Internal Medicine, McGill University, Montreal, CAN; 3 Endocrinology and Metabolism, Rutgers New Jersey Medical School, Newark, USA

**Keywords:** fna, cea, calcitonin, nodule, medullary thyroid cancer

## Abstract

Metastatic dissemination occurs in up to 90% of patients with medullary thyroid cancer (MTC) greater than 4 cm in diameter, and elevated calcitonin levels of more than 500 pg/ml preoperatively have been shown to predict the failure to achieve biochemical remission. Herein, we present a patient with a large tumor and extremely elevated calcitonin level, who was subsequently found to show a “benign” behavior with no evidence of metastasis. The relatively benign behavior of this patient's MTC despite its size and extremely elevated calcitonin levels is thought to be suggestive of certain mutation types in MTC to be more associated with better prognostic outcomes. This case report highlights the value of genetic studies on disease prognostication and the need for comprehensive research studies on genomic profiling in MTC to better understand the relationship of different mutations with prognosis and outcome.

## Introduction

Medullary thyroid cancer (MTC) is a neuroendocrine tumor of the parafollicular or C cells of the thyroid gland. MTC can be sporadic (75%) or familial (25%) and accounts for about 5% of thyroid cancers. The production of calcitonin is a characteristic feature of this tumor. Metastatic dissemination to both central and latero‐cervical lymph nodes occurs in up to 90% of patients with a tumor greater than 4 cm in diameter [1]. Calcitonin level can be used as a prognostic factor and an indicator of remission in MTC. Previous studies have shown that preoperative basal calcitonin level of more than 500 pg/ml is strongly associated with failure to achieve postoperative biochemical remission, which is defined by the failure of serum calcitonin to normalize and postoperative basal or stimulated calcitonin more than 10 pg/ml [2].

Herein, we present a patient with MTC who presented with extremely elevated calcitonin levels and was subsequently found to have a “benign” behaving type of cancer with no evidence of metastasis.

## Case presentation

**Figure 1:**
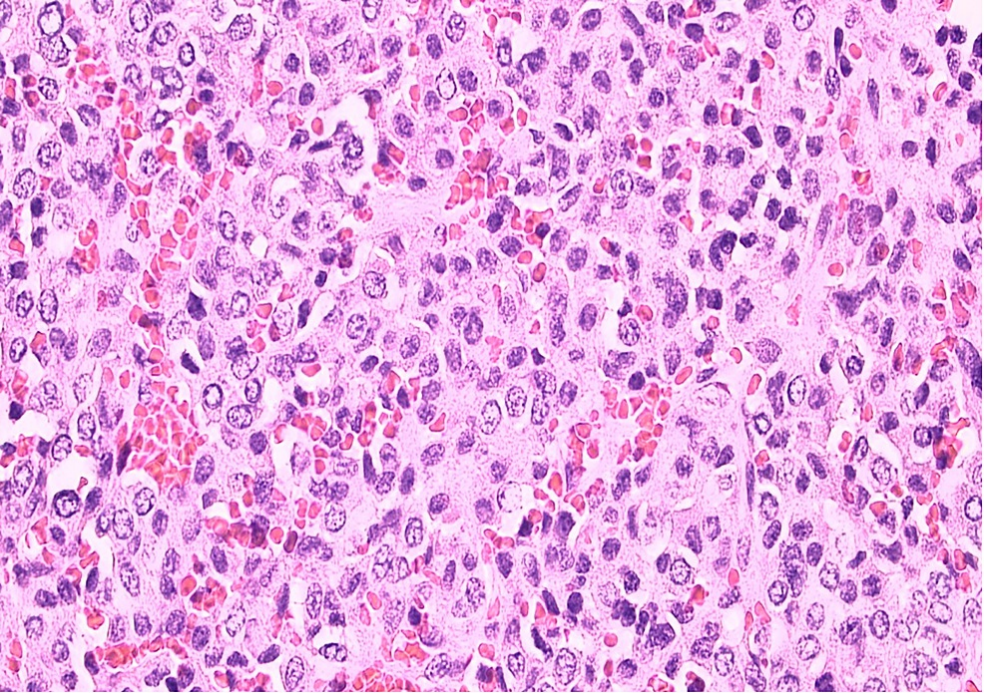
Shows solid sheets of epithelioid cells separated by a delicate fibrovascular stroma. The cytoplasm is amphophilic. The nuclei are round or oval with a salt and pepper chromatin pattern suggestive of a neuroendocrine origin of the tumor.

**Figure 2:**
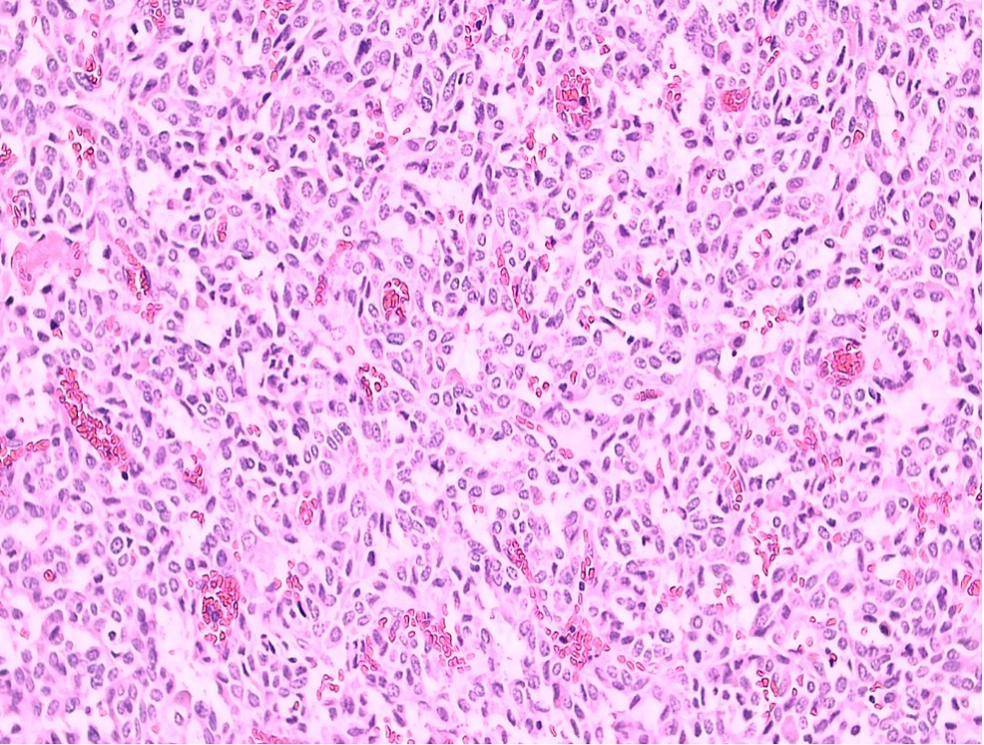
Shows round nuclei with finely stippled to coarsely clumped chromatin and indistinct nucleoli, which stains positive for amyloid on Congo red staining.

A 62-year-old woman presented to our endocrinology clinic for evaluation of a two-year history of “lump” sensation in the neck. She reported no dysphagia, choking sensation, neck pain, hoarseness, or any symptoms of hypo/hyperthyroidism. She had no history of radiation to the neck. Physical examination was only remarkable for a 4-cm thyroid nodule on the right side which moved easily with swallowing. No cervical lymphadenopathy was appreciated. Pemberton’s sign was negative. Ultrasound of the neck revealed a right-sided hypoechoic nodule measuring 4.12 x 1.84 cm. The thyroid function test was normal. Calcitonin and carcinoembryonic antigen (CEA) were 8754.0 pg/mL and 29.0 ng/mL, respectively. Fine-needle aspiration (FNA) cytology demonstrated a monomorphic population of numerous spindle cells with very few neoplastic cells that stained focally for Chromogranin A, consistent with MTC (Figures 1, 2).

Pan CT revealed no evidence of metastasis. The patient underwent total thyroidectomy after the exclusion of pheochromocytoma. Genetic studies were only remarkable for positive Harvey rat sarcoma viral oncogene homolog (HRAS) mutation. The result for rearranged during transfection (RET) mutation was negative, as were the results for paired box 8/peroxisome proliferator-activated receptor gamma (PAX8/PPARG), neuroblastoma RAS viral oncogene homolog (NRAS), and Kirsten rat sarcoma viral oncogene homolog (KRAS) mutation.

Following surgery, the patient was initiated on Levothyroxine to maintain TSH levels within normal limits. At 12 months of follow-up, calcitonin level declined to 3 ng/mL, and CEA remained within the normal range (1.5 ng/mL).

## Discussion

MTC is relatively an uncommon type of thyroid malignancy that arises from the neural crest-derived parafollicular C cells and accounts for approximately 5% or less of thyroid malignancies [3]. The discovery of MTC necessitates further investigations for evaluation of the disease extent, screening for pheochromocytoma and hyperparathyroidism, as well as genetic mutation analysis to determine whether MTC is sporadic or hereditary. Hereditary MTC accounts for about 25% of cases. Sporadic cases of MTC usually present with a palpable thyroid nodule and may arise clinically at any age with peak incidence during the fourth and sixth decades of life [1]. Prognostic values in MTC include age at diagnosis, gender, the initial extent of the disease based on lymph node involvement and distant metastases, vascular invasion, tumor size, and amyloid staining in tumor tissue. Lymph node metastases are found in up to 90% of MTC patients with a tumor greater than 4 cm in diameter. Distant metastases are usually diffuse and multiple in involved organs, including lymph nodes, liver, lungs, bones, and skin [1]. Additionally, measurement of serum calcitonin and carcinoembryonic antigen (CEA) have prognostic values in MTC. Serum calcitonin level plays an important role as a predictor for remission, the extent of surgery, and better arrangements for post-operative follow-up intervals. Calcitonin level above 500 pg/ml was found to have a strong indicative value for failure to achieve biochemical remission [2,4]. Also, assessment of calcitonin and CEA doubling times post-operatively provides sensitive markers for progression and aggressiveness of metastatic MTC [5].

MTC is characterized by activating RET proto-oncogene mutations in almost all hereditary cases (multiple endocrine neoplasia type 2A (MEN2A), multiple endocrine neoplasia type 2B (MEN2B), and familial MTC syndrome (FMTC)). Germline RET mutations can be indicative of hereditary MTC as well as predicting the lifetime risk for development of MTC, which can be near to 100% for mutation carriers. These mutations have been frequently detected in seemingly sporadic cases of MTC, which emphasizes the value of genetic testing in all MTC patients [6]. About 50% of sporadic cases of MTC have somatic RET mutations. Recent investigations have shown that 18%-80% of sporadic MTCs which lack somatic RET mutations have somatic mutations of HRAS, KRAS, and NRAS [7]. RAS mutations in MTC occur most commonly in HRAS (73.9% of all RAS mutations), followed by KRAS (23.1%) and NRAS (2.9%). These somatic RAS mutations have been noticed to be mutually exclusive with germline RET mutation, meaning that based on the existing data, if a somatic RAS mutation is found in association with MTC, then germline RET mutation can be considered unlikely [8]. Other studies have shown assessment of RAS mutation in sporadic RET-negative MTC can be valuable for personalized, targeted therapeutic strategies [9]. Reports suggest that MTC patients with HRAS Q61R mutations had an overall better survival rate and more favorable prognostic outcome [10].

## Conclusions

The relatively benign behavior of this patient's MTC despite the tumor size and significantly elevated calcitonin and CEA levels highlights the value of the results of genetic studies on disease prognostication. This case highlights the need for comprehensive research studies on genomic profiling in MTC to better understand the relationship of different genomic mutations with prognosis and outcome.
